# Widespread horizontal gene transfer between plants and bacteria

**DOI:** 10.1093/ismeco/ycae073

**Published:** 2024-05-13

**Authors:** Shelly Haimlich, Yulia Fridman, Hitaishi Khandal, Sigal Savaldi-Goldstein, Asaf Levy

**Affiliations:** The Department of Plant Pathology and Microbiology, Institute of Environmental Science, Robert H. Smith Faculty of Agriculture, Food, and Environment, The Hebrew University of Jerusalem, Rehovot 7610001, Israel; Faculty of Biology, Technion-Israel Institute of Technology, Haifa 3200003, Israel; Faculty of Biology, Technion-Israel Institute of Technology, Haifa 3200003, Israel; Faculty of Biology, Technion-Israel Institute of Technology, Haifa 3200003, Israel; The Department of Plant Pathology and Microbiology, Institute of Environmental Science, Robert H. Smith Faculty of Agriculture, Food, and Environment, The Hebrew University of Jerusalem, Rehovot 7610001, Israel

**Keywords:** horizontal gene transer, HGT, plant-microbe interactions, Arabidopsis thaliana evolution, microbiota, microbial evolution, DET2, brassinosteroid, auxin

## Abstract

Plants host a large array of commensal bacteria that interact with the host. The growth of both bacteria and plants is often dependent on nutrients derived from the cognate partners, and the bacteria fine-tune host immunity against pathogens. This ancient interaction is common in all studied land plants and is critical for proper plant health and development. We hypothesized that the spatial vicinity and the long-term relationships between plants and their microbiota may promote cross-kingdom horizontal gene transfer (HGT), a phenomenon that is relatively rare in nature. To test this hypothesis, we analyzed the *Arabidopsis thaliana* genome and its extensively sequenced microbiome to detect events of horizontal transfer of full-length genes that transferred between plants and bacteria. Interestingly, we detected 75 unique genes that were horizontally transferred between plants and bacteria. Plants and bacteria exchange in both directions genes that are enriched in carbohydrate metabolism functions, and bacteria transferred to plants genes that are enriched in auxin biosynthesis genes. Next, we provided a proof of concept for the functional similarity between a horizontally transferred bacterial gene and its Arabidopsis homologue *in planta*. The Arabidopsis *DET2* gene is essential for biosynthesis of the brassinosteroid phytohormones, and loss of function of the gene leads to dwarfism. We found that expression of the *DET2* homologue from *Leifsonia* bacteria of the Actinobacteria phylum in the *Arabidopsis det2* background complements the mutant and leads to normal plant growth. Together, these data suggest that cross-kingdom HGT events shape the metabolic capabilities and interactions between plants and bacteria.

## Introduction

Plants form intimate associations with microbes, collectively called the plant microbiota. Microbes mostly have commensal lifestyles with the plant. However, the microbial molecular mechanisms used to interface with the host molecular network are mostly elusive. Successful isolation and subsequent genome sequencing of hundreds of bacterial strains that are associated with diverse plant species and tissues enabled the elucidation of some of the microbial genes that are responsible for plant adaptation [1–6]. Transcriptome and proteome studies detected which bacterial genes are active *in planta* [7–9]. As part of the interaction between plants and their microbiota different molecules are being exchanged, including simple carbohydrates, organic acids, signaling molecules, antimicrobials, and bacterial effector proteins that change plant physiology [10–17].

One mechanism that may establish a stable interface between plants and their microbiota is cross-kingdom horizontal gene transfer (HGT) from the host to the microbe or vice versa. HGT provides the acceptor with the donor’s biological functions, such as new metabolic capacity and quick adaptation to the shared environment. The close proximity between the organisms, the selective pressure caused by immense microbial competition in the rhizosphere and by microbial interaction with the plant immunity, and the inherent ability of microbes to integrate foreign DNA may drive DNA transfer from plants into their microbiome [18]. Several plant-microbe HGT events were described. The pathogen *Pseudomonas syringae* acquired a eukaryotic E3 ubiquitin ligase domain as part of an effector protein AvrPtoB that degrades a host kinase, leading to host disease susceptibility [19]. The commensal *Bacillus subtilis* encodes a remote homologue of plant expansin proteins that was acquired through HGT. The bacterial expansin promotes plant cell wall extension and is critical for root colonization [20–22]. Gene transfer events from bacteria into plant germ cells have an unclear mechanism. However, such events were reported in the past and were described as mostly ancient HGT events from bacteria into the ancestors of land plants [23–26]. Several fern species contain an insecticidal chitin-binding protein that confers resistance to whitefly. It was suggested that the plant gene was acquired from bacteria as it has no close homolog in any other land plants, and the fern branch in the gene tree is nested within the bacterial branch [27, 28]. *Agrobacterium* spp. was shown to transfer tumor-inducing genes (T-DNAs) into the germline of different plant species including *Nicotiana*, *Linaria*, and *Ipomoea* [29]. A recent study estimated that bacteria were the most common gene donors to plants (much more than fungi and viruses), and identified two major episodes of HGT events corresponding to the early evolution of streptophytes and the origin of land plants [30].

We hypothesized that the genomes of plants and their root and shoot microbiome could uncover the extent and nature of cross-kingdom HGT events between plants and bacteria. In the current study, we performed a systematic search for potential cross-kingdom HGT events between the model plant *A. thaliana* and its extensively isolated and sequenced microbiome and bacteria which were isolated from different environments. Using phylogenetic analysis of proteins from 1766 organisms including plants, bacteria, archaea, animals, fungi, algae, SAR, and others, we could detect 75 HGT events between plants and bacteria. However, we could not detect a clear preference for HGT between plants and plant-associated (PA) bacteria. We could also detect 111 genes that were transferred between bacteria and eukaryotes (not exclusively to plants). In addition, we found that genes transferred from plants to bacteria are enriched in carbohydrate metabolism functions, such as cell wall degradation. In comparison, genes transferred from bacteria to plants are enriched in similar functions and also in auxin biosynthesis and response to hormones. Finally, we showed that a hormone biosynthesis gene transferred from plants to bacteria maintains its function *in planta* despite its divergence from the plant homologue through mutations. Our data suggest that cross-kingdom HGT events are frequent for plants and bacteria and likely facilitated efficient carbohydrate metabolism.

## Materials and methods

### Data sources and genome screening

Full genomic sequence of *A. thaliana* was downloaded from TAIR website (https://www.arabidopsis.org/). Additionally, a collection of 582 microbiota was prepared. These bacteria from the roots and shoots of *A. thaliana* were extensively isolated by different groups, and their genomes were previously sequenced. A list of 581 NPA bacteria was prepared following the PA bacteria list so that each class has a similar number of PA and NPA bacteria. The group of PA bacteria including 139 Actinobacteria, 271 Alphaproteobacteria, 49 Bacilli, 55 Betaproteobacteria, 1 Unknown, 1 Cytophagia, 13 Flavobacteria, 49 Gammaproteobacteria and 4 Sphingobacteria. When the group of NPA bacteria including 178 Actinobacteria, 188 Alphaproteobacteria, 57 Bacilli, 64 Betaproteobacteria, 3 Cytophagia, 18 Flavobacteria, 68 Gammaproteobacteria, and 5 Sphingobacteria.

Using BLASTP [65] version 2.8.1+ (standard settings), a comparison was made between the 582 PA bacteria and 581 NPA bacteria to Arabidopsis proteins. The hits were filtered of at least 35% amino acid sequence identity across > 80% protein length of the plant and the bacterial proteins. Protein sequences shared by bacteria and plant organelles, which themselves originate from bacteria, were filtered out. We filtered nuclear-encoded genes with association to plasticity and mitochondria in addition to plastid and mitochondria-encoded genes. Information on plant organelles origin is taken from ATH_GO_GOSLIM.txt file on the TAIR website (https://www.arabidopsis.org/download/GO and PO Annotations/Gene Ontology Annotations/ATH_GO_GOSLIM.txt. gz), In the “relationship type” column, we filtered out all the genes that are “located in” mitochondrion/chloroplast/etioplast/amyloplast/proplastid/chromoplast. At the end of this analysis, we identified 870 Arabidopsis proteins that were mapped to 110 315 bacterial proteins (Supplementary Table 2).

### Initial phylogenetic comparative methods to find HGT

A dataset that contains fully sequenced diverse organisms was created. The dataset contained 1191 genomes, including 1163 bacteria (582 PA bacteria, 581 NPA bacteria), 10 monocot and dicot plants, 8 fungi, 2 archaea, and 8 additional eukaryotes (Supplementary Table 1). Automatically, multiple sequence alignment was performed using Clustal Omega [66] version 1.2.4 using standard settings, and each of the 870 phylogenetic trees was constructed using FastTree [38] version 2.1.11 SSE3 using standard settings (Supplementary Data 1). Display, annotation, and management of phylogenetic trees were performed with Interactive Tree Of Life [67] (ITOL version 6.5).

Each of the phylogenetic trees was examined manually, and all the trees were divided into eight categories according to the inheritance pattern observed in the tree: (1) HGT from bacteria to plants, (2) HGT from plants to bacteria, (3) HGT from bacteria to eukaryotes, (4) HGT from eukaryotes to bacteria, (5) unclear phylogenetic, and (6) no HGT detection (Supplementary Table 3).

### Thorough phylogenetic comparative methods to find HGT

The genes from the aforementioned first four groups were examined in a stringent phylogenetic approach. Each group underwent comprehensive phylogenetic analysis, with the exception of the “HGT from bacteria to eukaryotes” group, for which a subset of 29 genes was randomly chosen for examination. Consequently, a total of 313 genes were tested again. For a comprehensive analysis of the phylogenetic trees, an additional 575 organisms were downloaded from the NCBI website with an “Assembly Level” of Complete Genome/Chromosome/Scaffold. The additional 575 organisms included 133 genomes of Amorphea (including animals, fungi, amoeba), 247 Archaea, 137 vascular plants, 18 Algae (green and red), 36 SAR (stramenopiles, alveolates, and rhizarians) clade genomes, 2 Bryophyta, and 2 Cryptista (Supplementary Fig. 4). Overall, genes from 1766 organisms were used in the analysis.

Multiple sequence alignment was performed using Clustal Omega [66] version 1.2.4 using standard settings, and each of the 313 phylogenetic trees was constructed using IQ-TREE [39] version 2.1.2 using customized settings (-mset LG,WAG,JTT,JTTDCMut -T AUTO -B 2000). The trees were rooted using MAD [40] (Supplementary Data 1). Display, annotation, and management of phylogenetic trees were performed with ITOL version 6.5 [67].

Each phylogenetic tree underwent a thorough manual examination to scrutinize evidence of HGT. When the tree included mostly bacterial genes and a bacterial branch shared a common ancestor with the plant branch, such trees were categorized as HGT from bacteria to plants. Conversely, in instances where the tree was predominantly eukaryotic and the plant branch shared a common ancestor with the bacterial branch, these trees were classified as HGT from plants to bacteria. Similar evaluations were conducted for transitions between bacteria and eukaryotes. We also inspected the ultrafast bootstrap approximation values (uf-bootstrap) to assess branch reliability. Trees wherein the branch shared by bacteria and plants/eukaryotes exhibited a uf-bootstrap value lower than 80 were denoted as having either a “Low bootstrap value” or “No bootstrap value,” as detailed in Supplementary Table 5.

### Gene ontology enrichment analysis

Functional information about the gene was performed using R packages clusterProfiler [68] on the Arabidopsis genes in four different groups: HGT from bacteria to plants, HGT from plants to bacteria, HGT from bacteria to eukaryotes, and HGT from eukaryotes to bacteria.

### Genomic neighborhood, structure, and function prediction and structure comparison

A pattern of genes present/absent was created by using the IMG website [69], using the option “Show neighborhood regions with the same top COG hit (via top homolog).” The structure prediction of the Arabidopsis proteins was downloaded from UniProt website (https://www.uniprot.org/), and the prediction structure of the bacterial protein was created using AlphaFold2 [70, 71] The structures comparison was created using PyMOL version 2.4.1 (https://pymol.org/2/). Structural similarity searches were done using the AlphaFold2 model of Arabidopsis proteins in UniProt and proteins were compared using Foldseek all protein databases [72].

### Graphs and figures

Various R packages are used to create graphs: ggplot2 [73], reshape2 [74], dplyr (https://dplyr.tidyverse.org), tidyverse (https://www.tidyverse.org/), and RColorBrewer (https://cran.r-project.org/web/packages/RColorBrewer/index.html).

**Figure 1:**
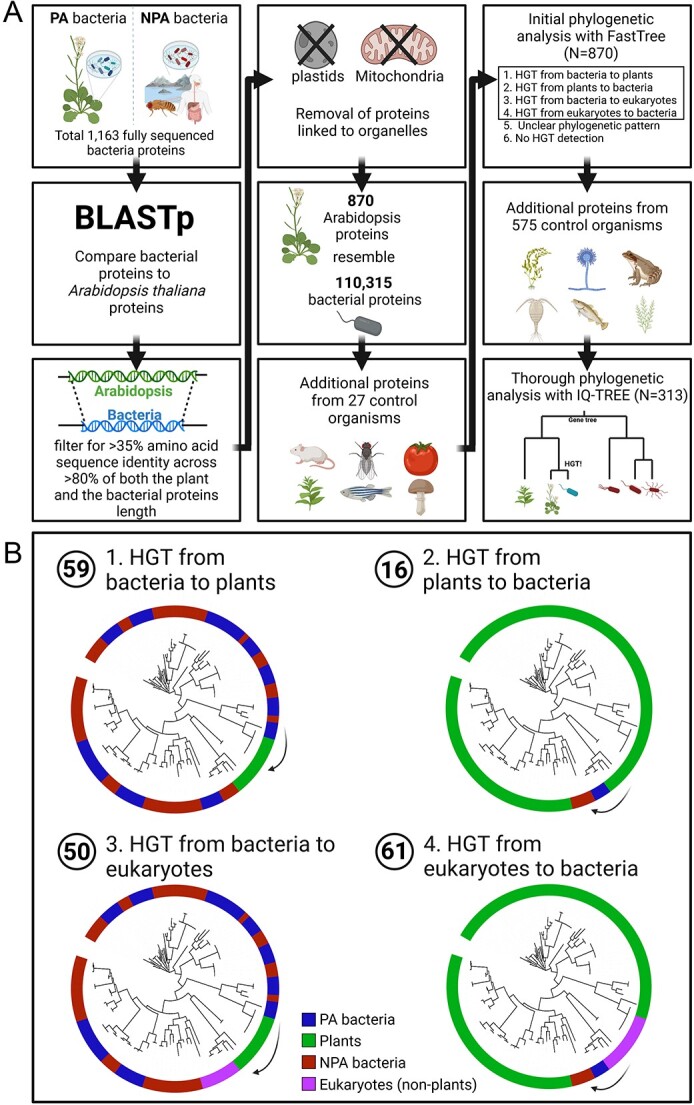
Analysis pipeline to detect cross-kingdom HGT events between bacteria and plants or eukaryotes in general. (A) An outline of the bioinformatic analysis we performed to detect cross-kingdom HGT events between plants and bacteria. Following comparison between proteins of 1163 bacteria and Arabidopsis we performed two rounds of phylogenetic analysis to detect HGT. The first analysis was performed with proteins from 27 additional control organisms, and yielded 246 putative HGT events between bacteria and plants. We improved this analysis by using a more stringent tree construction method with proteins from 575 additional organisms. Overall, genes from 1766 organisms were used in the analysis. (B) Classification of cross-kingdom HGT events into four different classes based on their predicted HGT directionality. Circles indicate the number of trees found from each category. PA bacteria, plant-associated bacteria; NPA bacteria, NPA bacteria.

BioRender (https://biorender.com/) was used to create Figs 1 and 3.

### Growth conditions, molecular cloning and transformation

For overexpression of *lfDET2*, the bacterial gene sequence underwent codon optimization for *A. thaliana*. The constructs were generated using the Golden Gate MoClo Plant Tool Kit [75]. For the DET2 promoter (*pDET2*), 550-bp fragment upstream to the first *DET2* ATG was used. *pDET2* and *p35S* in level 0 (in pICH41295) were then subcloned along with additional level 0 parts: lfDET2 (in pAGM1287), mNeonGreen (NG, in pAGM1301), and the *RBCS* terminator (in pICH41276) into level 1 (pICH47742). For overexpression of the Arabidopsis *DET2* (*atDET2*), a similar cloning procedure was used except that the *DET2* terminator was used. The constructed level 1 was then subcloned into a level 2 construct (pAGM4723), together with a level 1 kanamycin resistance gene (pICH47732). Plant transformation to wild-type (WT) Col-0 and *det2* backgrounds was performed using the *Agrobacterium tumefaciens* (GV3101)-mediated floral dip transformation method. Transgenic lines were screened on selective 0.5 MS plates supplied with 50 mg/l kanamycin (Duchefa Biochemie). Homozygous lines were selected according to mendelian segregation of the selection marker. For each construct used, two to three independent transgenic lines were generated. Presented here are line 6 (*det2;pDET2:lfDET2*), line 3 (*det2;p35S:lfDET2*), and line 4 (*pDET2:atDET2*)*.* Plant growth conditions were as described by Fridman et al. [76]. Briefly, seeds were surface sterilized and germinated on one-half-strength (0.5) Murashige and Skoog (MS) medium supplemented with 0.8% plant agar, 0.46 g/l MES pH 5.8, 0.2% (w/v) sucrose. Plates with sterilized seeds were stratified at 4°C for 2 days in the dark before transfer to the growth chamber with 16-h light/8-h dark cycles, at 22°C. Irradiance conditions of ∼70 μmol m^−2^ s^−1^.

### Confocal microscopy

Confocal microscopy was performed using a Zeiss LSM 510 (Zeiss, Jena, Germany) confocal laser scanning microscope with a LD LCI Plan-Apochromat 25x water immersion objective (NA-0.8), or LSM 710 (Zeiss, Jena, Germany) using a Plan-Apochromat 20x objective (NA-0.8). Roots were imaged in water, or with water supplemented with propidium iodide (PI, 10 μg/mL). The green fluorescent proteins NeonGreen (NG) and PI were excited by an argon laser (488 nm) and by DPSS laser (561 nm), respectively. For PI detection in LSM 710, solid state laser (543 nm) was used. In LSM 510, fluorescence emission signals for NG and for PI were collected by PMT detectors, with a band-pass filter (500–530 nm) and a long-pass filter (575 nm), respectively. In LSM 710, fluorescence emission signals for NG and for PI were collected by BIG detectors (GaAsP) with a band-pass filter (500–550 nm) and a band-pass filter (570–620 nm), respectively.

### lfDET2 sequence after codon optimization

ATGCCCGACGGTCCGTATCGCTGGTTCGTGTATGCCGAGATCGCCCTCGCGGTGGTCACCTTCGTCGCTCTGTGCTTCGTGGTAGCGCCGTACGGACGGCACGGCCGCTCCGGATGGGGGCCGACCGTGCCCGCGCGGGTCGGCTGGGTCGTGATGGAGAGTCCAGCATCCATCGTCTTCCTGCTGTTCTACCTGCTCGGCGACCACCGGTTCGAGCTGGTGCCTCTGCTGTTCCTCGCGCTGTGGCAGCTCCACTACGTGCAGCGTGCCTTCGTCTACCCGTTCCTGATGCGCACCGGGTCCAGGATGCCCGTGTCCGTCGTGGGGATGGCGATCCTGTTCAACCTGCTCAACGCGTGGGTGAATGCGCGGTGGATCTCGCAGTACGGCCAGTACGCGAACAGCTGGCTCGCCGACCCTCGGTTCTGGATCGGCGTGGTCGTGTTCATCGCCGGGTTCTCGCTCAACCTCGGTTCCGACCGCATCCTGCGCAGACTGCGGGGTGCGCGATCCGGCGGGTACAGCATCCCGCGCGGTGGCGGATACCGCTGGGTGTCCAGCCCGAACTACCTGGGCGAGATGGTGGAGTGGACCGGCTGGGCGATCGCGACCTGGTCGCTCGCCGGGCTGGCGTTCGCGCTGTACACGATCGCGAACCTCGCACCGCGGGCGATGGCGAACCACCGCTGGTACCTGGAGACGTTCGACGACTATCCGCCGGAGCGAAAAGCGATCATCCCCTATCTGCTCTGA.

## Results

### Identification of genes that share high amino acid sequence similarity between *A. thaliana* and its microbiome

To quantify the extent of HGT events between plants and microbiome we focused on the plant *A. thaliana* that serves as a model for plant-microbe interactions [31, 32]. The microbiota of Arabidopsis have been extensively isolated from roots and shoots by different groups and their genomes were previously sequenced [1, 2, 4]. We compared the genes of *A. thaliana* against the genes of 582 fully sequenced PA bacteria that were isolated from *A. thaliana* (Supplementary Table 1). Our current analysis includes commensal Arabidopsis-associated bacteria which were isolated from roots and shoots of healthy plants in the US, Germany, and Switzerland. As a control group, we used 581 non-plant associated (NPA) bacteria, which were isolated from diverse non-plant environments such as animals and aquatic environments (Supplementary Table 1). The NPA bacteria group does not contain bacteria that were isolated from soil which may also colonize plants. To serve as a control group, a list of 581 NPA bacteria was prepared following the PA bacteria list so that each class has a similar number of PA and NPA bacteria. PA and NPA bacteria belong to Actinobacteria, Proteobacteria, Firmicutes, and Bacteroidetes phyla, and their classification to PA and NPA is based on their original isolation site, which we previously manually curated [2]. We used the BLASTp program to detect identity between the set of 48 149 proteins encoded by Arabidopsis, 2 909 309 proteins from PA bacteria, and 2 489 944 proteins from NPA bacteria (Fig. 1a). We focused on identity between full-length protein sequences, as these proteins are relatively poorly studied in comparison to horizontally transferred protein domains, which are extensively studied in the context of effectors of pathogenic bacteria [33, 34]. These sequences were later subjected to an extensive phylogenetic analysis to suggest the existence of HGT based on inconsistency within gene trees (see below). According to some definitions two protein sequences are considered homologous if they are > 30–35% identical over their entire lengths [35, 36]. Therefore, we used only BLASTP hits of at least 35% amino acid sequence identity across at least 80% of the coverage of both the Arabidopsis and the bacterial proteins.

Next, we filtered out what we termed “trivial hit,s” which are protein sequences shared by bacteria and plant organelles, i.e. mitochondria and chloroplast, which themselves originate in bacteria [37], and their proteome may maintain homology to their original bacterial proteome. This analysis resulted in a list of 870 *Arabidopsis* proteins that are mapped to 110 315 bacterial proteins and likely play similar functions (Fig. 1A, Supplementary Table 2). One concern is that protein sequence similarity-based search will result in detection of Arabidopsis or bacterial DNA contaminants that were mistakenly assembled in bacterial or Arabidopsis genomes, respectively. However, we did not identify bacterial-Arabidopsis homologous protein pairs with amino acid identity above 76%, rejecting the possibility of hypothetical DNA contamination that was introduced during the genome assembly process, which would result in highly similar protein sequences.

### Phylogenetic analysis leads to detection of 75 genes that demonstrate cross-kingdom horizontal transfer between plants and bacteria

The proteins we identified as sharing high sequence identity between plants and bacteria can be the result of various evolutionary scenarios. For example, the genes can be ancient and conserved between eukaryotes and prokaryotes. We specifically searched for phylogenetic evidence supportive of direct gene transfer between plants and bacteria, with a focus on the plant microbiota. To this end we compiled a dataset that contained 1191 genomes (Supplementary Table 1), including the aforementioned 1163 PA and NPA bacteria, 10 monocot and dicot plants, 8 fungi, 2 archaea, and 8 additional eukaryotes (animals, parasites, and a mold).

For each of the 870 Arabidopsis genes with a high resemblance to the PA or NPA bacteria genes, we constructed a phylogenetic gene tree, using FastTree [38], based on multiple sequence alignment of all gene hits within our genome dataset (Supplementary Data 1). We defined an event of cross-kingdom HGT from plants to bacteria or vice versa when a subset of organisms from both groups shared the same branch in the gene tree demonstrating an inconsistency between the genome tree and the gene tree (Fig. 1B). Specifically, we verified that the branch that is composed of homologues from plants and bacteria did not include homologues from animals or fungi. Similarly, we also defined HGT between plants and PA bacteria (or NPA bacteria as control) to examine whether there is an enrichment in the gene transfer between plants and their microbiota, compared to bacteria from other environments. To increase accuracy, we ignored cases where the transfer occurred between plants and only a single bacterium to reject the possibility of hypothetical DNA contamination.

We detected 246 HGT events between bacteria and plants. Nonetheless, we suspected that some of these were false positives due to inaccurate phylogenetic analysis. To provide additional support for the detection of HGT events, we used a more stringent phylogenetic approach applied to 313/870 trees from the previous stage, including all 246 events in which we detected HGT between plants and bacteria and for 67 of the cases in which we detected HGT between bacteria and eukaryotes. We added to the phylogenetic analysis 575 additional control eukaryotic and prokaryotic genomes to fill missing gaps in the evolutionary trees, used the more accurate and rigorous IQ-TREE method [39] for phylogenetic tree reconstruction, and rooted the trees using MAD [40]. The additional 575 organisms included 133 genomes of Amorphea (mostly animals, fungi, amoeba), 247 Archaea, 137 vascular plants, 18 Algae (green and red), 36 SAR (stramenopiles, alveolates, and rhizarians) clade genomes, 2 Bryophyta, and 2 Cryptista, (Supplementary Fig. 4). Indeed, this stringent phylogenetic analysis led to discarding of most previous HGT calls.

We determined the direction of the HGT by examining the organisms within the tree leaves. For example, in case the tested gene was present in most of the examined bacteria and was present in only the plant group within the eukaryotic domain, nested within a bacterial branch, it is more parsimonious to assume that the gene was transferred from bacteria to plants and no massive deletion occurred in all the eukaryotes in our dataset. However, we cannot rule out more complex evolutionary scenarios. We defined the donor domain (e.g. bacteria) based on the organisms in the closest sister clades of the branch that contains plants and bacteria.

Overall, we identified 59 genes that were likely transferred from bacteria to plants (Supplementary Table 5, Supplementary Figs 1–3). For example, homologues of gene AT4G24350 are found in multiple bacterial branches and in one of them there is a branch, nested within a bacterial branch, and contains only genes from vascular plants (Supplementary Fig. 1). Nearly all these genes are ancient and are conserved in nearly all vascular plants and at least one of the groups Bryophyta or algae (Supplementary Table 5). In this group, we found five HGT events that are from PA bacteria to plants (Table 1) and three HGT events that are from NPA bacteria to plants. One of the genes transferred from PA bacteria to plants is ARR17, which is responsible for sex determination in poplar trees [41] and acts as a cytokinin response regulator [42]. We also identified 16 genes that were likely transferred from plants to bacteria (Supplementary Table 5, Supplementary Figs 4–6). In this group, we found two HGT events from plants to PA bacteria (Table 1) and six HGT events from plants to NPA bacteria. The genes that were transferred into PA bacteria are the homologues GH9C2 and GH9C3, encoding endoglucanase glycosyl hydrolases [43]. It is clearly observed that only a small number of bacterial genomes acquired the GH9C2 gene from plants (Supplemental Fig. 4). GH9C2 and GH9C3 are present in four (three Bacillaceae and one Streptomycetaceae) and five (three Bacillaceae, one Sphingomonadaceae, and one Streptomycetaceae) bacterial genomes, respectively.

**Table 1:** Genes exchanged between PA bacteria and plants.

**Arabidopsis locus**	**Phylogenetic pattern**	**Arabidopsis gene symbol**	**Arabidopsis gene full name**	**Arabidopsis gene description**	**Predicted protein function based on structure prediction**
AT2G35810	HGT from PA bacteria to plants	Unavailable	Unavailable	Ureidoglycolate hydrolase	Ureidoglycolate hydrolase/lyase (particpates in purine metabolism)
AT2G35830	HGT from PA bacteria to plants	Unavailable	Unavailable	Ureidoglycolate hydrolase	Ureidoglycolate hydrolase/lyase (particpates in purine metabolism)
AT3G13180	HGT from PA bacteria to plants	AtTRM4e, TRM4e	tRNA methyltransferase 4e	NOL1/NOP2/sun family protein/antitermination NusB domain-containing protein	RNA methytransferase, binds RNA
AT3G56380	HGT from PA bacteria to plants	RR17, ARR17	Response regulator 17	Response regulator 17	Two component response regulator
AT4G17085	HGT from PA bacteria to plants	Unavailable	Unavailable	Putative membrane lipoprotein	
AT1G64390	HGT from plants to PA bacteria	AtGH9C2, GH9C2	Glycosyl hydrolase 9C2	Glycosyl hydrolase 9C2	Glycoside hydrolase family 9
AT4G11050	HGT from plants to PA bacteria	AtGH9C3, GH9C3	Glycosyl hydrolase 9C3	Glycosyl hydrolase 9C3	Glycoside hydrolase family 9

Overall, 75 existing Arabidopsis unique proteins were horizontally transferred to or acquired from bacteria based on our phylogenomic analysis, in addition to organelle genes, 79% of which were acquired by plants. Considering the number of gene transfers between plants and PA or NPA bacteria, these findings refute our hypothesis that plant microbiota, or at least the microbiota of *A. thaliana*, are more likely to donate or acquire plant genes than other control (NPA) bacteria, despite the close plant-microbiome proximity. In general, the genes transferred to or from PA bacteria are poorly studied with relatively little information about their functions. It would be interesting to study what has led to fixation of these plant genes in bacterial genomes.

In addition, we identified 50 genes that were horizontally transferred from bacteria to the eukaryotic domain (Supplementary Table 5, Supplementary Figs 7–9). For example, RNR1 (AT2G21790) is a prokaryotic gene, present in bacteria and archaea, that was transferred into a large number of eukaryotes, including many plants (Supplementary Fig. 8). We found two HGT events that are from PA bacteria to eukaryotes and one HGT event that is from NPA bacteria to eukaryotes. Additionally, we identified 61 genes that were horizontally transferred from eukaryotes to bacteria (Supplementary Table 3, Supplementary Figs 10–12). For example, MIOX4 (AT4G26260) is a bacterial gene that was transferred to bacteria from Amorphea (Supplementary Fig. 12). In this group, we found seven HGT events from eukaryotes to PA bacteria and three HGT events from eukaryotes to NPA bacteria.

### Genes that were transferred between plants and bacteria are enriched in carbohydrate metabolism processes

We wondered if certain gene functions are more likely to undergo cross-kingdom HGT due to an adaptive function conferred to the acceptor organism. To address this question we performed a functional enrichment analysis of the genes that have been horizontally transferred (Materials and Methods).

**Figure 2:**
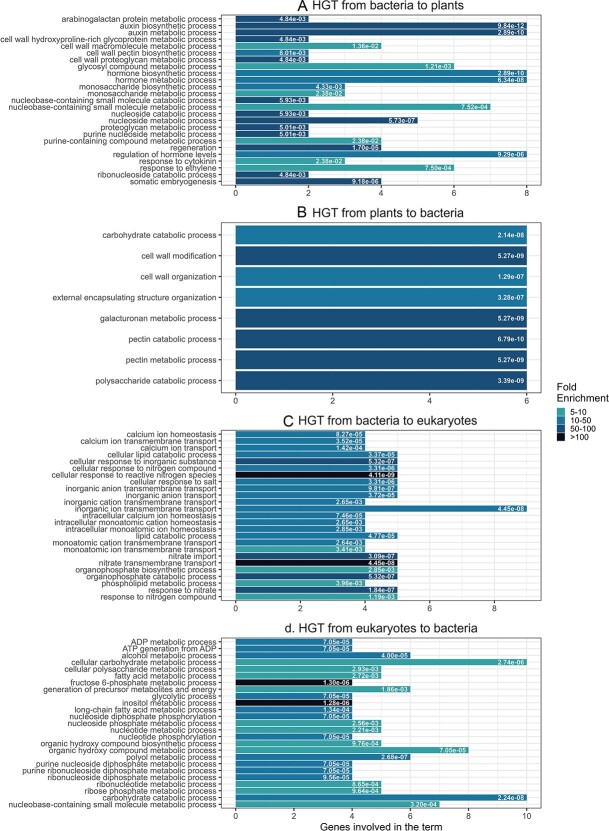
Gene ontology (GO) enrichment analysis of the different groups that have signatures of HGT. GO enrichment analysis using R packages clusterProfiler. The 25 most significantly (*P* < 0.05, after false discovery rate correction) enriched GO terms. q-values are printed on the bars. (A) GO functional analysis of Arabidopsis genes (*n* = 59) with an HGT pattern from bacteria to plants. (B) GO functional analysis of Arabidopsis genes (*n* = 16) with an HGT pattern from plants to bacteria. (C) GO functional analysis of Arabidopsis genes (*n* = 50) with an HGT pattern from bacteria to eukaryotic domain. (D) GO functional analysis of Arabidopsis genes (*n* = 61) with an HGT pattern from eukaryotic domain to bacteria.

The genes transferred from bacteria to plants are enriched in genes encoding auxin biosynthesis, nucleoside metabolism, and glycosyl compound metabolism (Fig. 2A). The glycosyl compound meatbolism proteins include for example 3-Deoxy-D-manno-octulosonate 8-phosphate synthase, aldolase like protein (AT4G24080), alpha-amylase-like, Sugar isomerase (SIS) family proteins (AT5G52190, AT5G42740), and glycosyl hydrolase family protein 43. Auxin biosynthesis genes that were acquired from bacteria included many of the YUCCA genes: YUC1, YUC3, YUC5, YUC6, YUC7, YUC8, YUC9, and YUC11. Previous works also suggested that these genes were acquired from bacteria, as a consequence of plant interaction with microbes [24, 44]. Another gene acquired from bacteria is IAMH2: an Indole-3-acetamide hydrolase gene that is required for the auxin effects of Indole-3-acetamide [45].

The genes transferred from plants to bacteria are strongly enriched in carbohydrate catabolic processes (*P* = 2.14 × 10^−8^, 6 out of 16 transferred genes) (Fig. 2B). The transferred genes encode enzymes like pectin esterase or glycosidase activity and they likely target the plant cell wall or exploit carbohydrates from the root exudates [46]. These genes include, for example, various genes encoding chitinases (e.g. CHI, AT2G43570), glycosyl hydrolases such as an endo beta mannanase (XCD1, AT3G10890) and glycosyl hydrolase 9C2 (GH9C2, AT1G64390, see Supplemental Fig. 4), pectin lyases (AT1G05310, AT1G11370, AT5G07420, AT5G61680), and pectin methylesterases (AT3G29090, AT5G47500). The chitinase (CHI) gene also serves as a defense gene that is induced in plants during Systemic Acquired Resistance [47, 48] and its transfer to bacteria may be used to manipulate plant defense response or to directly degrade chitin from fungi or insects that are present in the plant environment.

Genes transferred from bacteria to eukaryotes are enriched in functions related to inorganic transmembrane transport (Fig. 2C), and genes transferred from eukaryotes to bacteria are enriched in carbohydrate catabolism (Fig. 2D), such as the endo-beta-mannanase family.

### Signatures of recent HGTs into bacterial genomes can be detected

**Figure 3:**
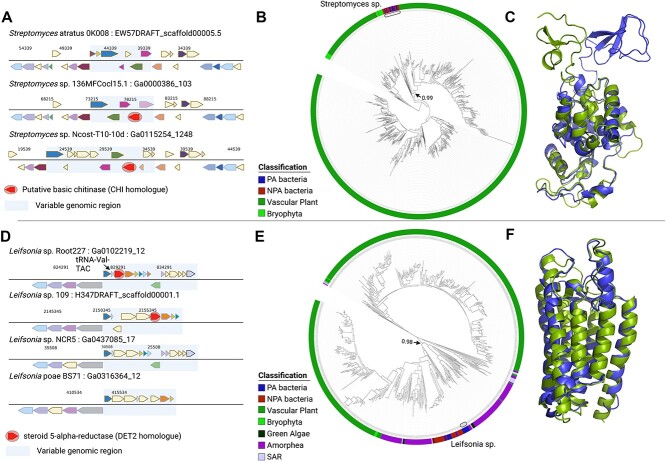
Examples of likely recent HGT from plants or other eukaryotes to bacteria. (a) Pattern of the presence/absence of the CHI homologue gene (marked with a circle) in PA *Streptomyces* genomes. (b) A phylogenetic tree that presents CHI protein found mainly in plants and also found in a small group of PA *Streptomyces*. The bootstrap value of the clade that is shared by plants and their bacteria is 0.99 (marked with an arrow). (C) Structure comparison of CHI plant protein (green) and bacterial protein (blue), RMSD = 0.791. The top part represents the N-terminus. (D) Pattern of presence/absence of the DET2 homologue gene (marked with a circle) in *Leifsonia* genomes. (E) A phylogenetic tree that presents DET2 proteins found mainly in the eukaryotic domain and in a small group of Actinomycetia, including *Leifsonia* bacteria. The bootstrap value of the clade that is shared by plants and their bacteria is 0.98 (marked with an arrow). (F) Structure comparison of DET2 plant protein (green) with bacterial protein (blue), RMSD = 0.727.

In several cases, we identified trans-kingdom HGT events into bacteria that likely occurred relatively recently. These events were characterized by insertion into a narrow bacterial taxon. In addition, through the inspection of the genomic neighborhood of the acquired gene we observed that the gene had a patchy presence/absence pattern between members of the same genus and was located in a relatively variable genomic region compared to its flanking regions. One example is the bacterial homologue of the plant-specific *CHI* gene (AT2G43570), encoding a putative basic chitinase. *CHI* gene is a defense gene that is strongly upregulated in plants in response to butterfly oviposition [49]. The *CHI* gene is present mostly in *Streptomyces* genomes (22/25 bacterial genomes) but not in other Actinobacteria that we analyzed, suggestive of a relatively recent gene acquisition/loss event (Fig. 3B). Not even all *Streptomyces* genomes encode the *CHI* gene (note *Streptomyces atratus* OK008 in Fig. 3A). Comparison of the predicted protein structures of the plant and bacterial CHI homologues demonstrate a striking similarity with root-mean-square-deviation (RMSD) = 0.791 (Fig. 3C). The N-terminus of CHI protein presented the largest difference between the two structures.

Another interesting example is of the bacterial homologue of *DET2,* a steroid-5-alpha-reductase which is one of the key genes in the brassinosteroid biosynthetic pathway [50]. *DET2* is the only gene from the brassinosteroid biosynthetic pathway that we detected in bacteria. The plant *DET2* gene is shared with other eukaryotes from the Amorphea taxonomic supergroup (animals, fungi, amoeba, choanoflagellates) and there are some bacterial orthologues of this gene (Fig. 3E), all of which are from the Actinomycetia class (phylum Actinobacteria). Interestingly, within the bacterial kingdom the plant *DET2* is most closely related by sequence to bacterial *DET2* orthologs encoded by the *Leifsonia* genus from Actinomycetia class. The Arabidopsis DET2 protein is 46% identical to the *Leifsonia* Det2 homologue. Strikingly, this level of sequence identity is shared between the Arabidopsis DET2 and its homologues from rice and barley. Importantly, the DET2 proteins from other eukaryotic plant pathogens such as the oomycetes *Phytophthora* and *Pythium* share weaker identity (maximum 41% identity) to the Arabidopsis protein than the *Leifsonia*-Arabidopsis DET2 similarity. Given the strong plant-bacteria protein identity, we suggest a member of the Actinomycetia class was a direct gene acceptor of a eukaryotic gene, and the donor was a member of Amorphea and not a plant. The bacterial gene is located in a patchy distribution (namely, present in only some genomes) within a variable genomic region downstream to a tRNA gene (Fig. 3D) that may mediate foreign DNA integration into the locus [51]. Although the plant and bacterial DET2 homologues share <50% sequence identity their predicted structures are strikingly similar with RMSD = 0.727, suggestive of a similar biochemical function (Fig. 3f).

### Horizontally transferred bacterial gene can functionally replace its homologous plant gene

**Figure 4:**
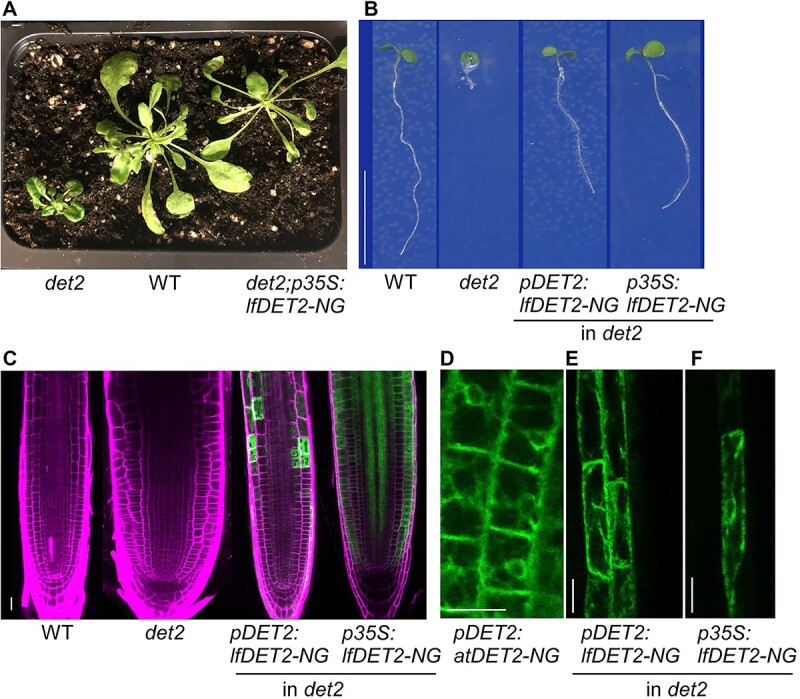
Complementation of *det2* Arabidopsis by a *det2* bacterial homologue reveals functional similarity between bacterial and Arabidopsis homologous genes. A comparison between WT, *det2*, and transgenic *det2* lines harboring *pDET2:lfDET2-NG* and *p35S:lfDET2-NG* (*det2* expressed from two promoters). (A) Adult developmental stage of *det2*, WT, and *p35S:lfDET2-NG*. Note the WT-like phenotype of the rescued *det2*. (B) 7-day-old seedlings of WT, *det2*, *pDET2:lfDET2-NG*, and *p35S:lfDET2-NG.* Scale bar = 1 cm c. root meristem of lines as in (A). Note the wide and aberrant morphology of the *det2* meristem and its rescue by lfDET. mNeonGreen(NG) is shown in green and propidium iodide (PI) that marks cell borders in magenta. (D–F) Subcellular localization of atDET2 and lfDET2 in epidermal root cells. Note their similar localization in the endoplasmic reticulum. Scale bars = 20 um.

We tested if the bacterial genes we identified as being horizontally transferred from eukaryotes can replace their homologous plant genes. As a proof-of-concept we selected *DET2* (Fig. 3D–F). The *A. thaliana det2* mutant has a severe dwarf phenotype including a wider root meristem with altered cell wall orientation, typical to brassinosteroid (BR) deficient mutants (Fig. 4A–C) [52, 53]. We transformed this mutant background with *Leifsonia* Det2 (lfDET2) fused to a fluorescent protein (lfDET2-NG), driven by the constitutive 35S or the Arabidopsis DET2 promoters and observed a rescue of these BR phenotypic defects (Fig. 4A–C). The rescued root length remained slightly shorter than WT, similar to an equivalent transformation with the Arabidopsis DET2 (*atDET2*) [54]. In agreement with the atDET2-like functionality *in planta*, lfDET2 localized to the endoplasmic reticulum, similar to *atDET2* (Fig. 4D–F). To conclude, lfDET2 is functionally similar to its Arabidopsis homologue.

## Discussion

In this work we looked closely on the effect of HGT on the evolution of plants and bacteria, with a focus on *A. thaliana* and its extensively sampled microbiome. The effect of HGT on plant evolution was described in several works in recent years. Analysis of the moss *Physcomitrella patens* identified 44 families of nuclear genes that were acquired from bacteria in comparison to only 12 gene families that were acquired from either fungi, archaea or viruses [24]. These findings include two gene families that are involved in auxin biosynthesis. Green plants acquired from bacteria genes related to biosynthetic and metabolic pathways, adaptation, exaptation, and stress response [55]. These include, for example, genes involved in xylan degradation, plant vascular system development, and stress response to cold and cadmium. Genome analysis of subaerial Zygnematophyceae algae concluded that gene families that increase resistance to biotic and abiotic stresses in land plants, in particular desiccation through abscisic acid (ABA), were acquired by HGT from soil bacteria [56]. A recent work analyzed HGT patterns in 12 representative plant species [30]. The authors identified two major HGT episodes in plant evolution, each of which contributed > 100 gene families. The first occurred during early evolution of streptophytes and the second at the origin of land plants. Most of the contributing organisms were described as bacteria, with some contribution from fungi. These results also support our current results that nearly all transfers into Arabidopsis are ancient and are shared by monocots and dicots. We could not detect a bacterial gene that was transferred directly to Brassicaceae and is absent from other dicots.

Previous works could not specifically determine which microbes were gene donors or acceptors. We reasoned that the microbiome of a plant is the natural partner for HGT with the plant. We focused on bacteria and not on fungi and archaea because there is a high number of sequenced Arabidopsis-associated bacteria and because previous works showed that they are the most common gene donors to plants [24]. Another innovation of our method is the separation between bacteria according to their isolation sites to PA and NPA with the latter serving as a control group. This distinction allowed us to suggest a direct bi-directional HGT path between plants and their microbiome that affected at least seven genes (Table 1). However, interestingly, we could not observe evidence that this path is more common than HGT between plants and NPA bacteria which do not share a niche. We do not know the precise mechanism of HGT between plants and NPA bacteria. One explanation could be that some PA organisms (bacteria or eukaryotes) that are missing from our analysis served as the mediators to transfer the genes to plants or NPA bacteria. An alternative would be that these genes are ancient and have undergone gene loss except in plants and NPA bacteria. Previously, it was reported that HGT occurs much more frequently between bacteria that share the same ecological niche (e.g. inhabit the same human body site) than between bacteria from different niches [57]. However, our data indicate that shared ecology of a host and its microbiome is not a critical indicator for cross-kingdom HGT, at least in the case of *A. thaliana* serving as a host. It would be interesting to test this finding in the context of other plants, animals or humans and their microbiomes. Some animal-bacteria cross-kingdom HGT events were described before such as the citrullinating genes that were transferred to animals from Cyanobacteria [58], peptidoglycan biosynthesis genes that were transferred into mealybugs [59] and peptidoglycan degradation genes acquired by the deer tick [60]. Another finding from our analysis is that we identified much fewer plant to bacteria gene transfer events (*n* = 16) than in the opposite direction (*n* = 59). This is, in our view, quite surprising as bacteria are much more amenable than plant cells to acquiring genes from the environment via transformation of environmental DNA, combined with a much faster generation time to allow natural selection and gene fixation.

The observed enrichment of carbohydrate catabolism functions, specifically of plant sugars such as pectin, which have moved from plants to bacteria, is fascinating. These genes, including the genes for pectin lyases and methylesterases, increased plant-dependent bacterial growth and likely the ability to colonize plant tissues by breaking down the plant cell wall. Pectate lyases enzymes were also shown to be critical for endophytic Arabidopsis root colonization by fungi, and they reduced plant performance [61]. Interestingly, in a previous study we revealed that genomes of PA bacteria have a higher number of carbohydrate metabolism genes than the genomes of the NPA group from the same taxon [2]. This trend was reproducible when we examined groups of bacteria from four different phyla. In the current work, we propose a model that actually a small number of these genes were transferred directly from plants to their microbiome. For example, we identified that glycosyl hydrolase 9C2 and 9C3 were directly transferred into PA bacteria.

Several phytohormone pathways have previously been described as transferred between plants and associated microbes. *Agrobacterium* naturally transfers its auxin biosynthesis genes into its host plant [62]. It was suggested that the YUC genes that are involved in Auxin biosynthesis were transferred from bacteria to the most recent common ancestor of land plants [24]. Agrobacterium also encodes and transfers into plants genes required for production of cytokinin [63]. On the other hand, as part of the interaction with plants, rhizobia evolved an independent pathway for gibberellin production [64]. In the current work, we suggest that *det2*, a gene from the brassinosteroid biosynthetic pathway, has laterally transferred from eukaryotes to bacteria from the *Leifsonia* genus, including several PA bacteria. We showed that the gene can replace its plant homologue in brassinosteroid production but the role of this gene in bacteria remains unknown. We performed Arabidopsis root colonization experiments with *Leifsonia* strains that are naturally positive or negative for *det2* and could not detect a phenotypic effect that correlates with the gene presence, at least when Arabidopsis were grown on agar. Together, our results suggest that cross-kingdom HGT shaped the genomes of both plants and bacteria, and that specific gene functions were acquired by bacteria, likely to break down the unique set of sugars that plants are capable of producing.

## Supplementary Material

Supplementary_material_ycae073

## Data Availability

All data generated or analysed during this study are included in this published article and its supplementary information files.
