# Supplementary material for: Widespread horizontal gene transfer between plants and bacteria
Source: ISME Commun. 2024 May 13;4(1):ycae073. doi: 10.1093/ismeco/ycae073 (PMC11131428; doi:10.1093/ismeco/ycae073)
Supplement: Supplementary_material_ycae073 [file supplementary_material_ycae073.zip › Supplementary Information (4).pdf]

Supplementary Information

Widespread horizontal gene transfer between plants and bacteria

Shelly Haimlich[1], Yulia Fridman[2], Hitaishi Khandal[2], Sigal Savaldi-Goldstein[2], * ,and Asaf Levy[1]

The department of plant pathology and microbiology, Institute of [1] Environmental Science, Robert H. Smith Faculty of Agriculture, Food, and Environment, The Hebrew University of Jerusalem, Rehovot 7610001, Israel.

[2] Faculty of Biology, Technion-Israel Institute of Technology, Haifa 32000, Israel

Corresponding author *

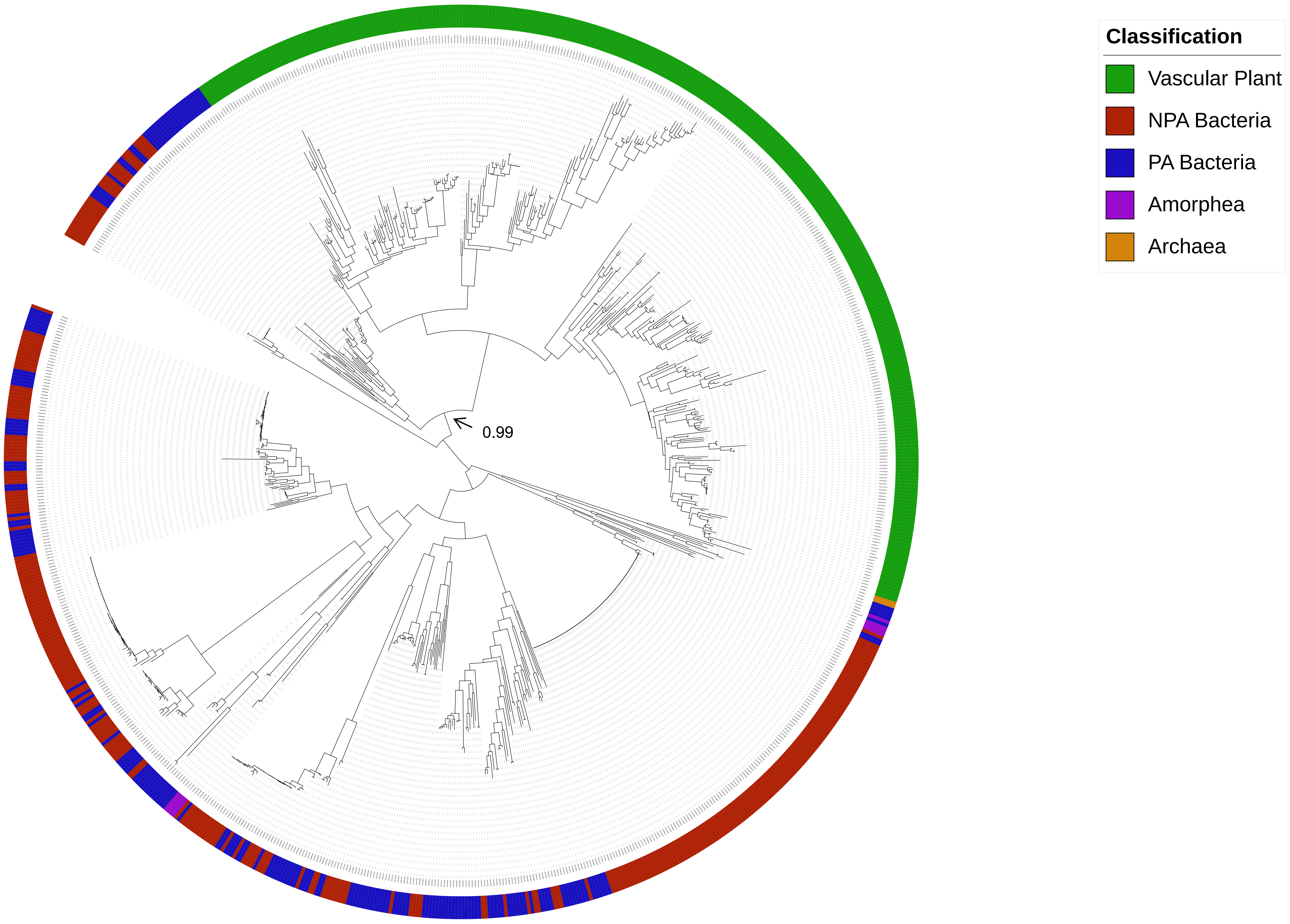

Supplemental Figure 1. Example of HGT from bacteria to plants. A phylogenetic tree that presents homologs of the AT4G24350 gene. The ultrafas bootstrap value of the clade that is shared by plants and bacteria is 0.99 - marked with an arrow

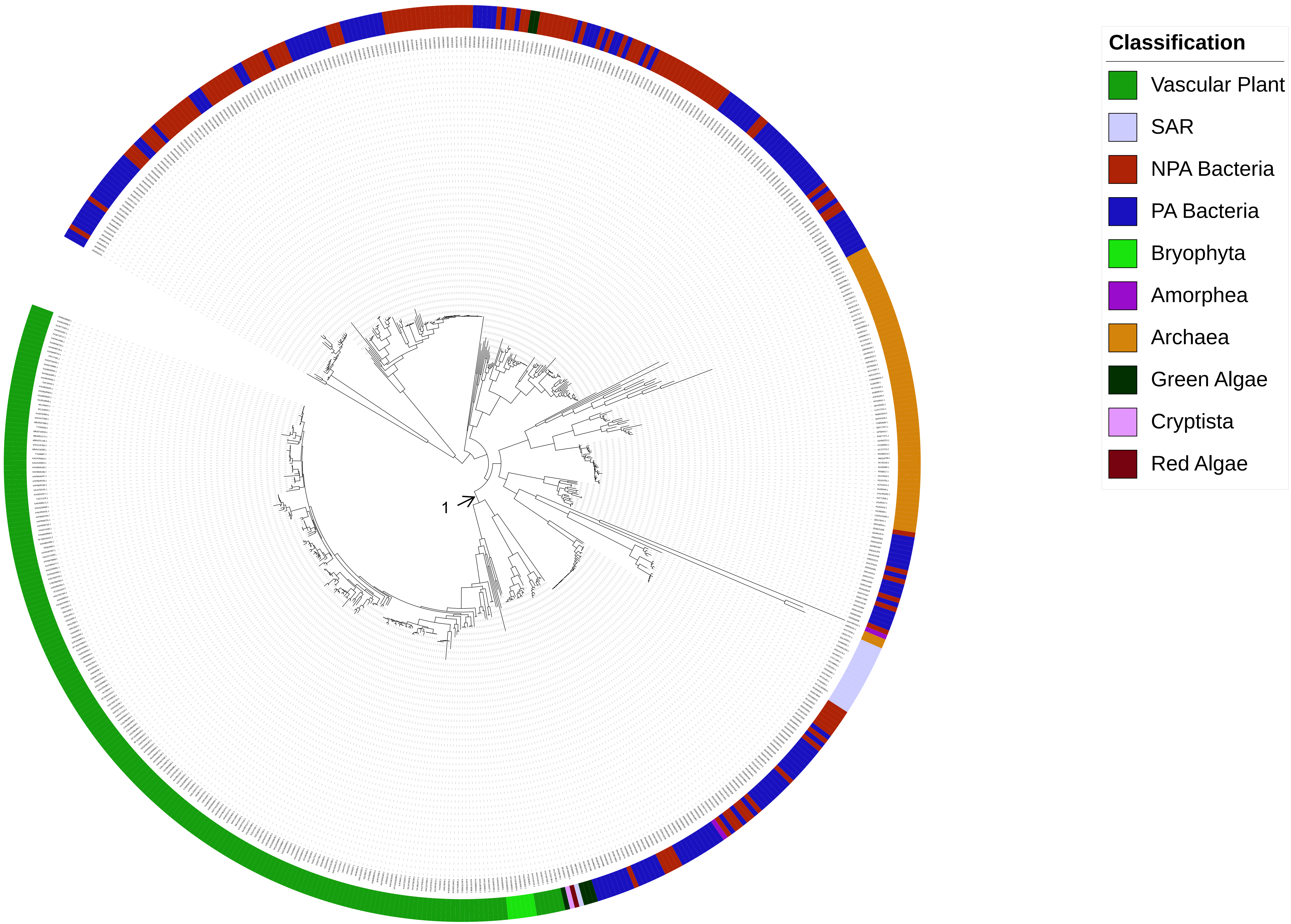

Supplemental Figure 2. Example of HGT from bacteria to plants. A phylogenetic tree that presents homologs of the AT1G54310 gene. The ultrafas bootstrap value of the clade that is shared by plants and bacteria is 1 - marked with an arrow

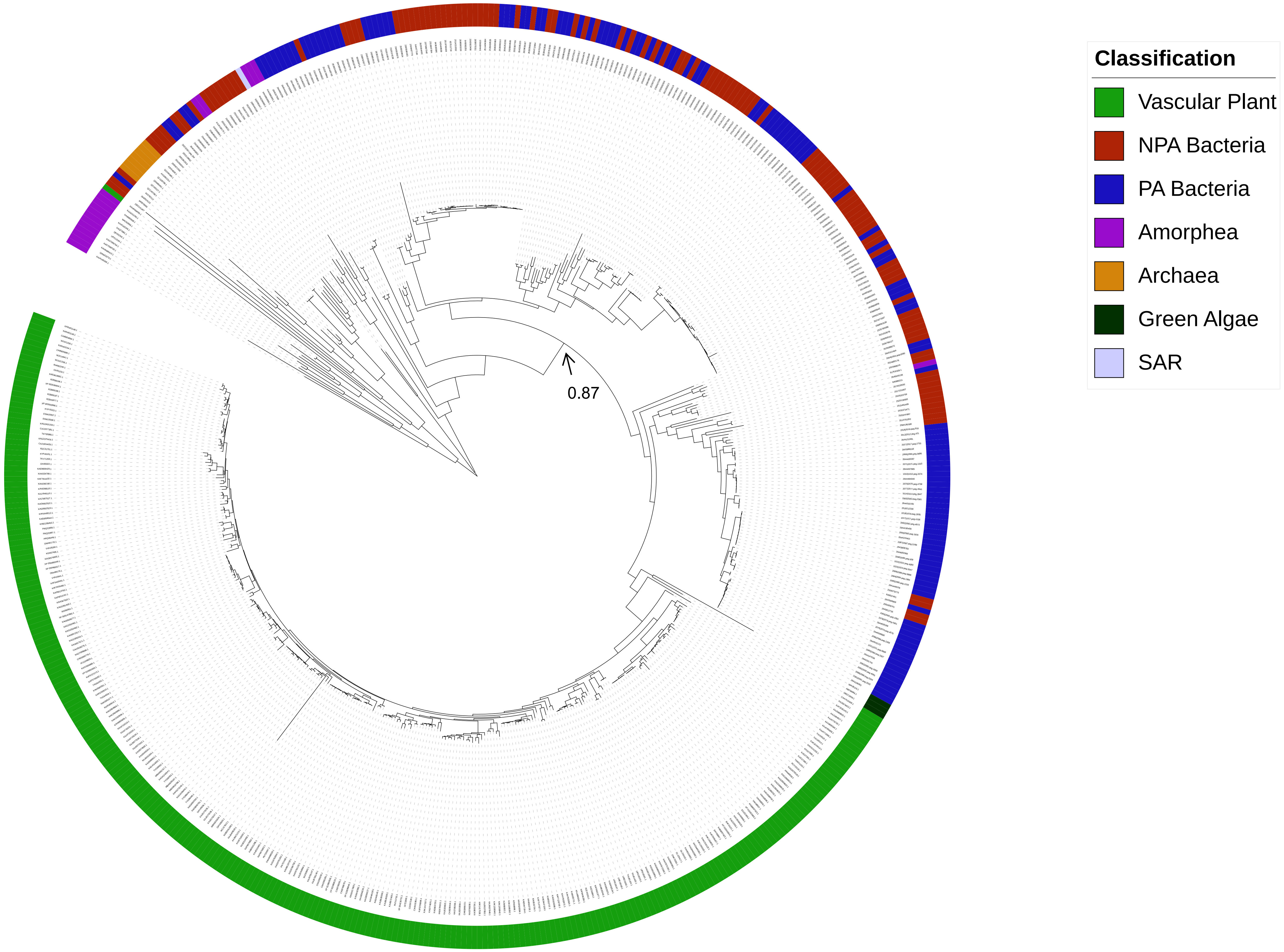

Supplemental Figure 3. Example of HGT from bacteria to plants. A phylogenetic tree that presents homologs of the MTK (AT1G49820) gene. The ultrafas bootstrap value of the clade that is shared by plants and bacteria is 0.87 - marked with an arrow

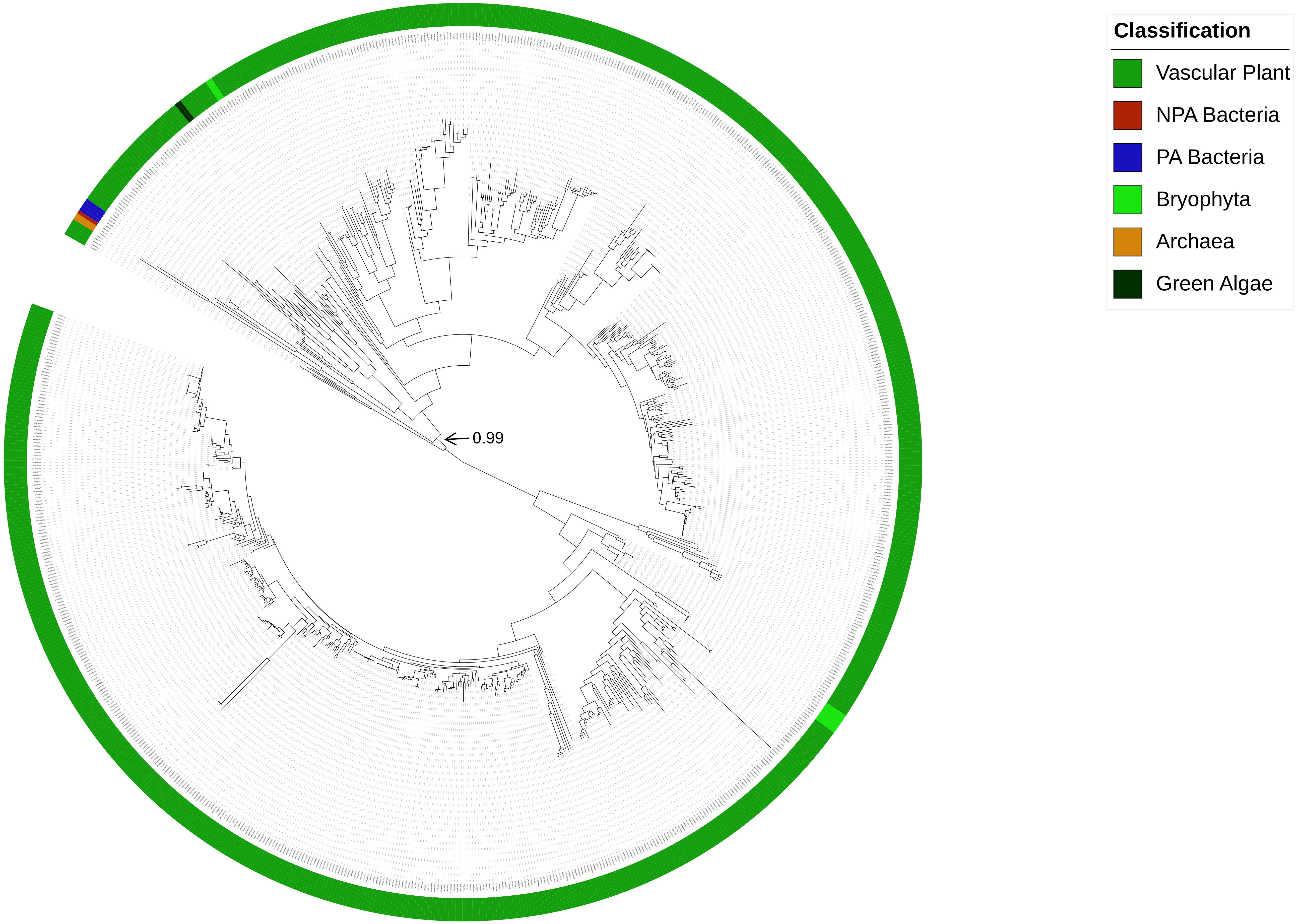

Supplemental Figure 4. Example of HGT from plants to bacteria. A phylogenetic tree that presents homologs of the GH9C2 (AT1G64390) gene. The ultrafas bootstrap value of the clade that is shared by plants and bacteria is 0.99 - marked with an arrow

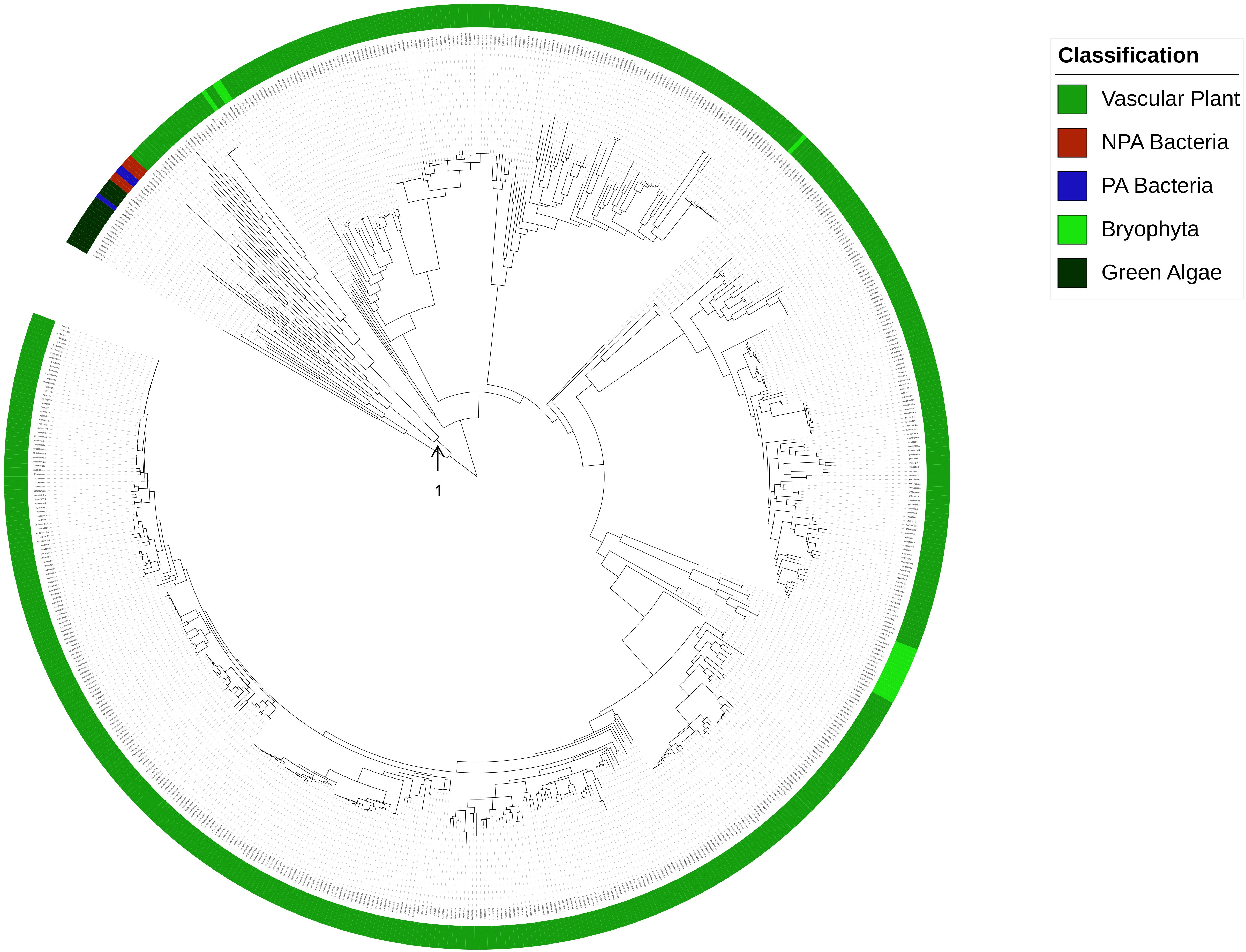

Supplemental Figure 5. Example of HGT from plants to bacteria. A phylogenetic tree that presents homologs of the SLP3 (AT2G19170) gene. The ultrafas bootstrap value of the clade that is shared by plants and bacteria is 1 - marked with an arrow

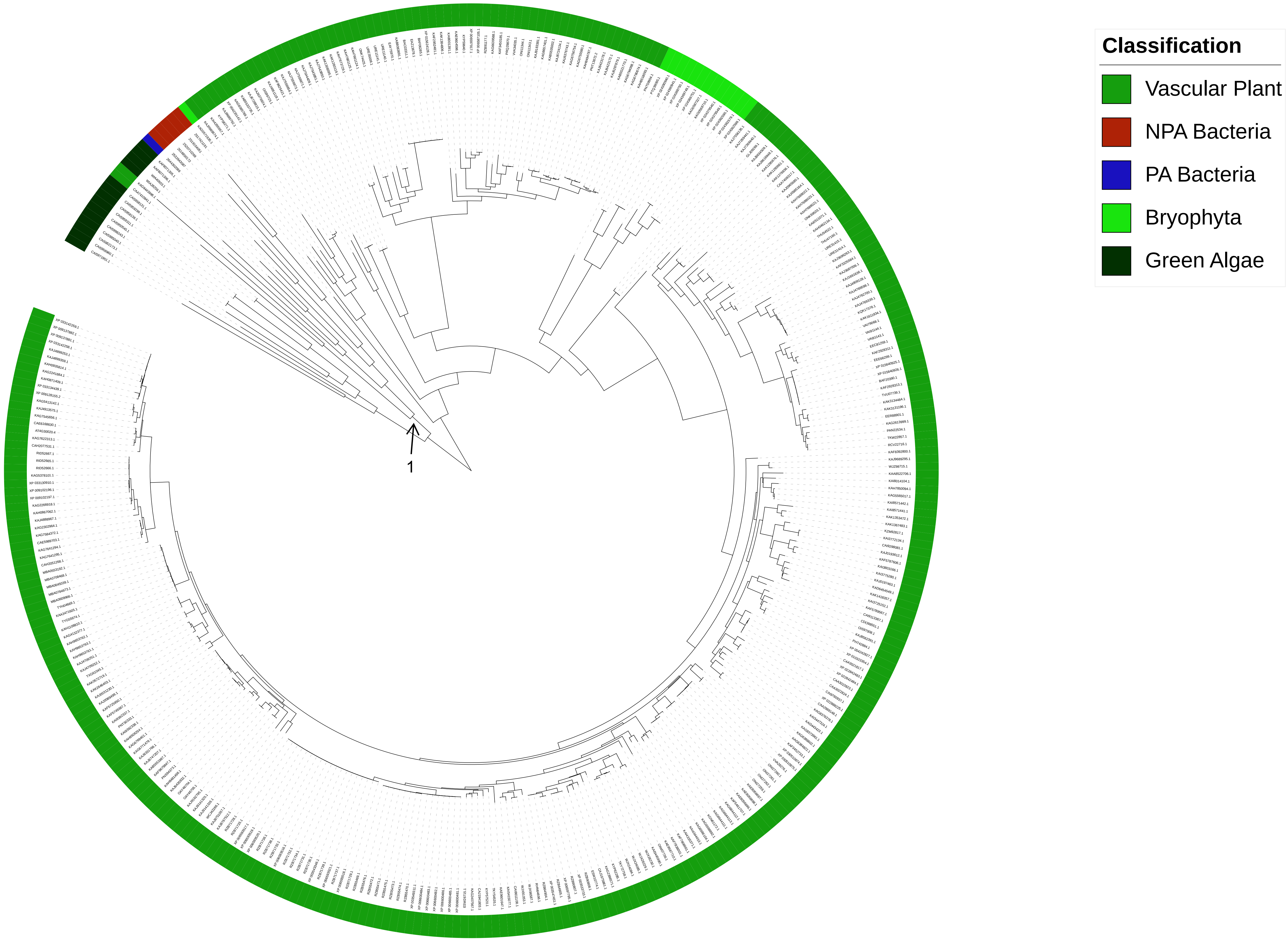

Supplemental Figure 6. Example of HGT from plants to bacteria. A phylogenetic tree that presents homologs of the AT4G30020 gene. The ultrafas bootstrap value of the clade that is shared by plants and bacteria is 1 - marked with an arrow

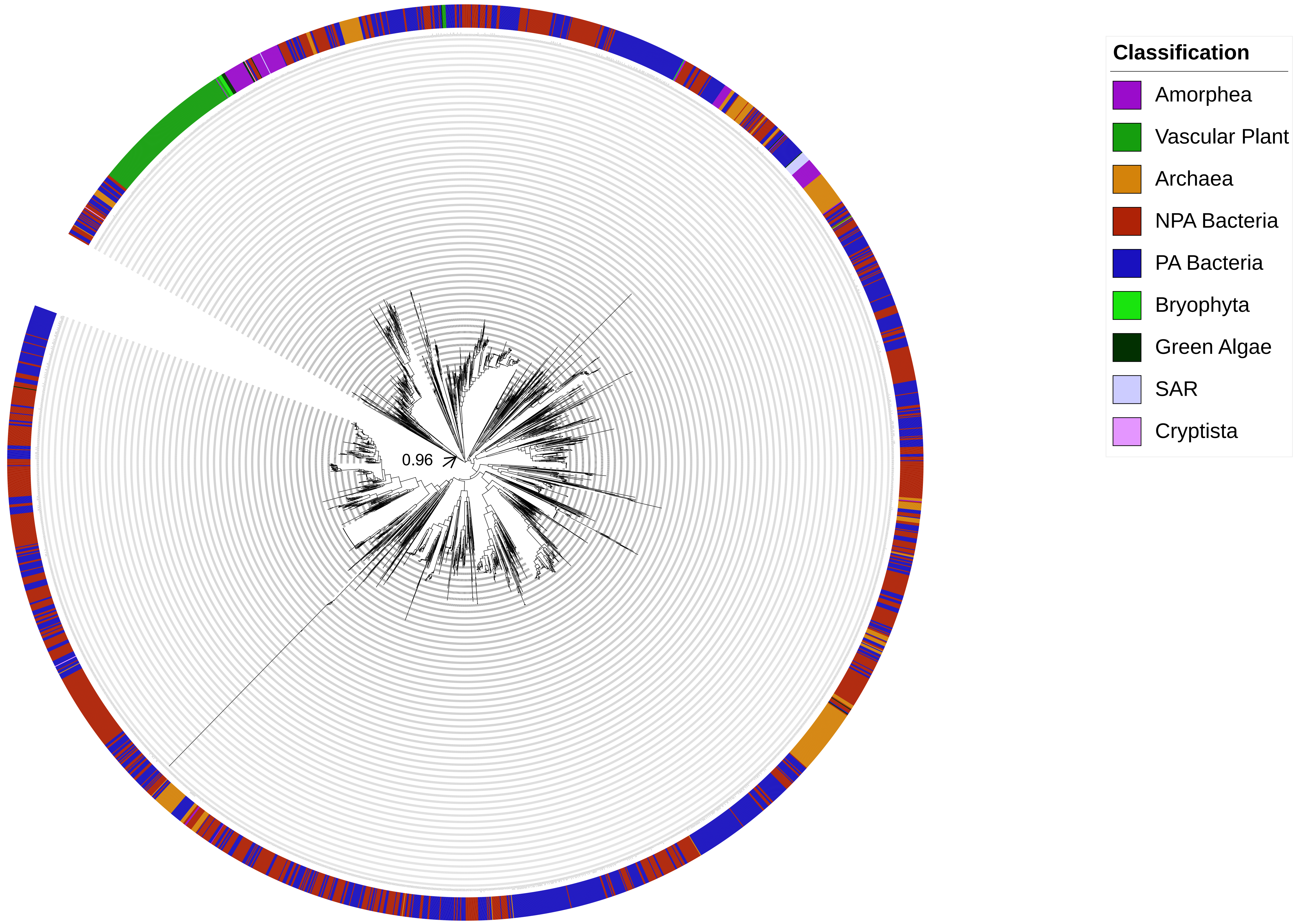

Supplemental Figure 7. Example of HGT from bacteria to eukaryotes. A phylogenetic tree that presents homologs of the NUDX1 (AT1G68760) gene. The ultrafas bootstrap value of the clade that is shared by eukaryotes and bacteria is 0.96 - marked with an arrow

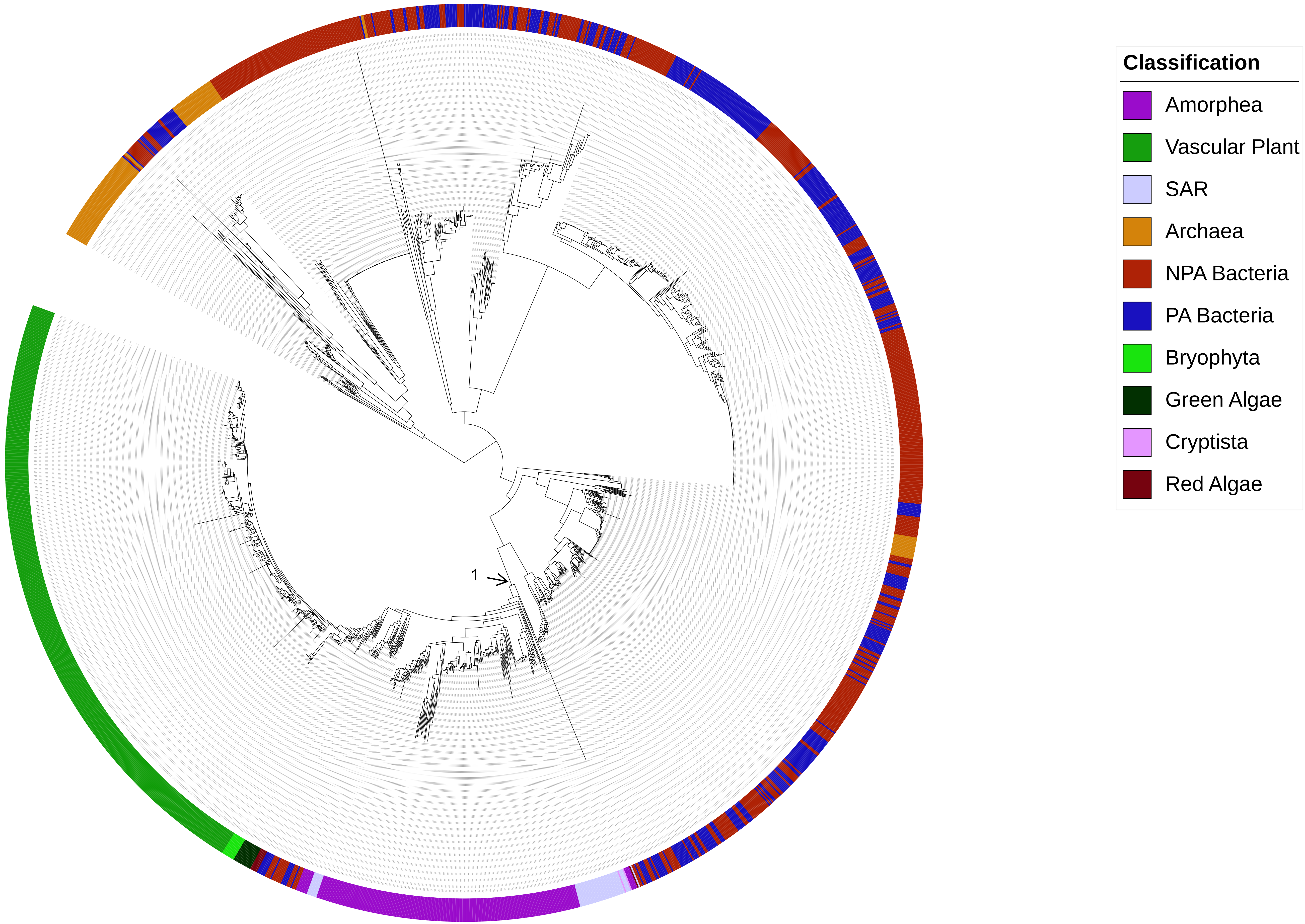

Supplemental Figure 8. Example of HGT from bacteria to eukaryotes. A phylogenetic tree that presents homologs of the RNR1 (AT2G21790) gene. The ultrafas bootstrap value of the clade that is shared by eukaryotes and bacteria is 1 - marked with an arrow

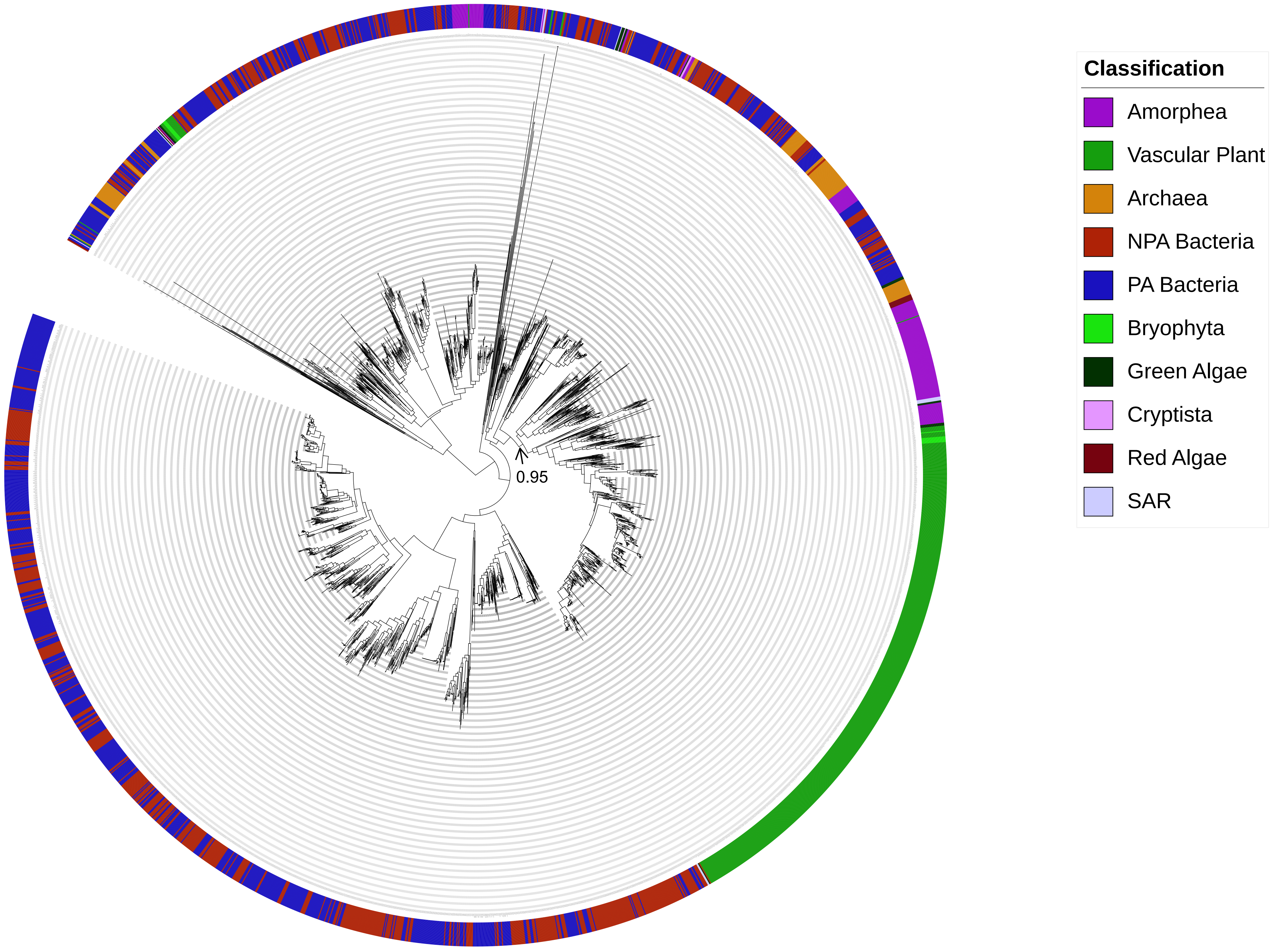

Supplemental Figure 9. Example of HGT from bacteria to eukaryotes. A phylogenetic tree that presents homologs of the PRN (AT3G59220) gene. The ultrafas bootstrap value of the clade that is shared by eukaryotes and bacteria is 0.95 - marked with an arrow

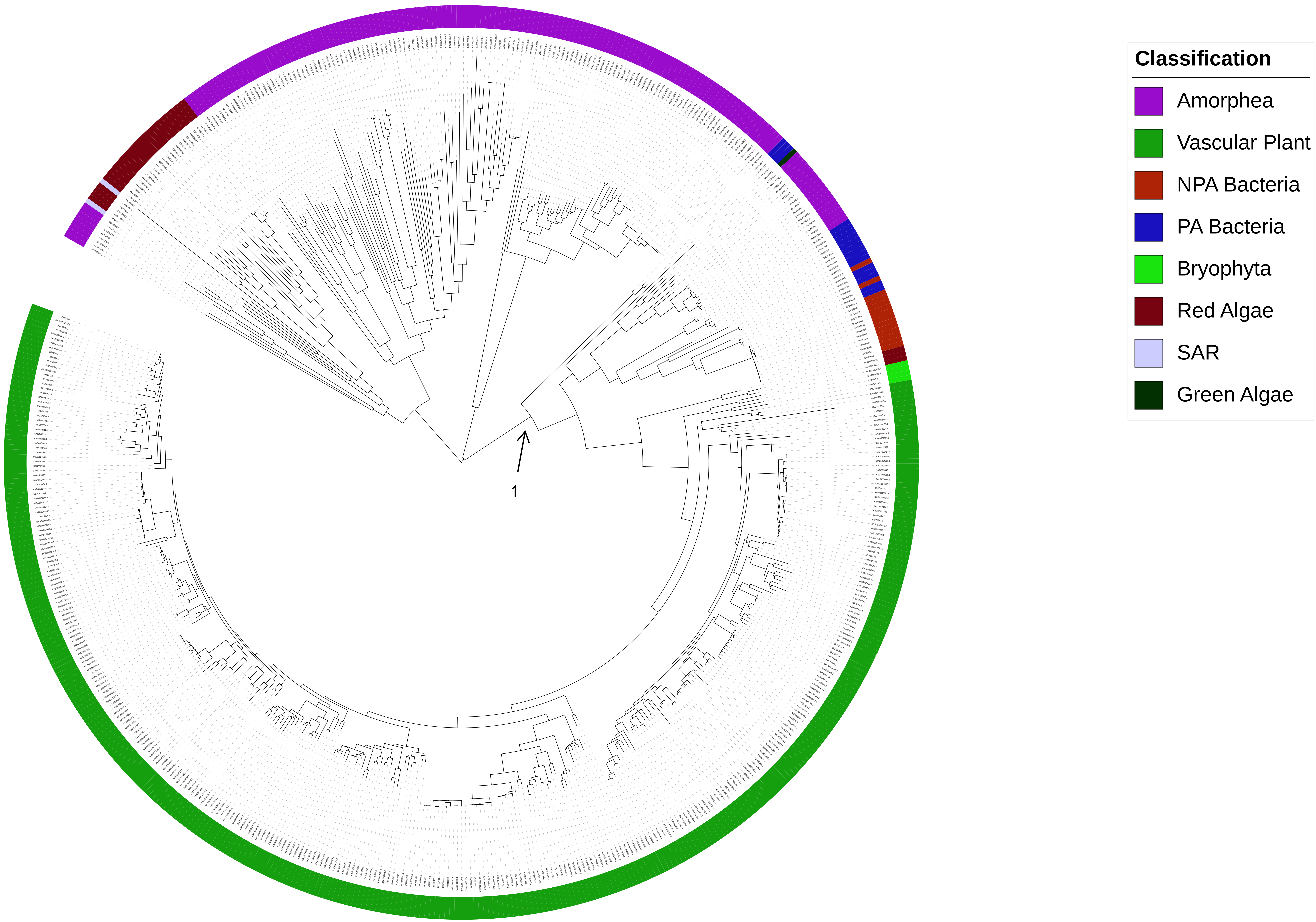

Supplemental Figure 10. Example of HGT from eukaryotes to bacteria. A phylogenetic tree that presents homologs of the DOX1 (AT3G01420) gene. The ultrafas bootstrap value of the clade that is shared by eukaryotes and bacteria is 1 - marked with an arrow

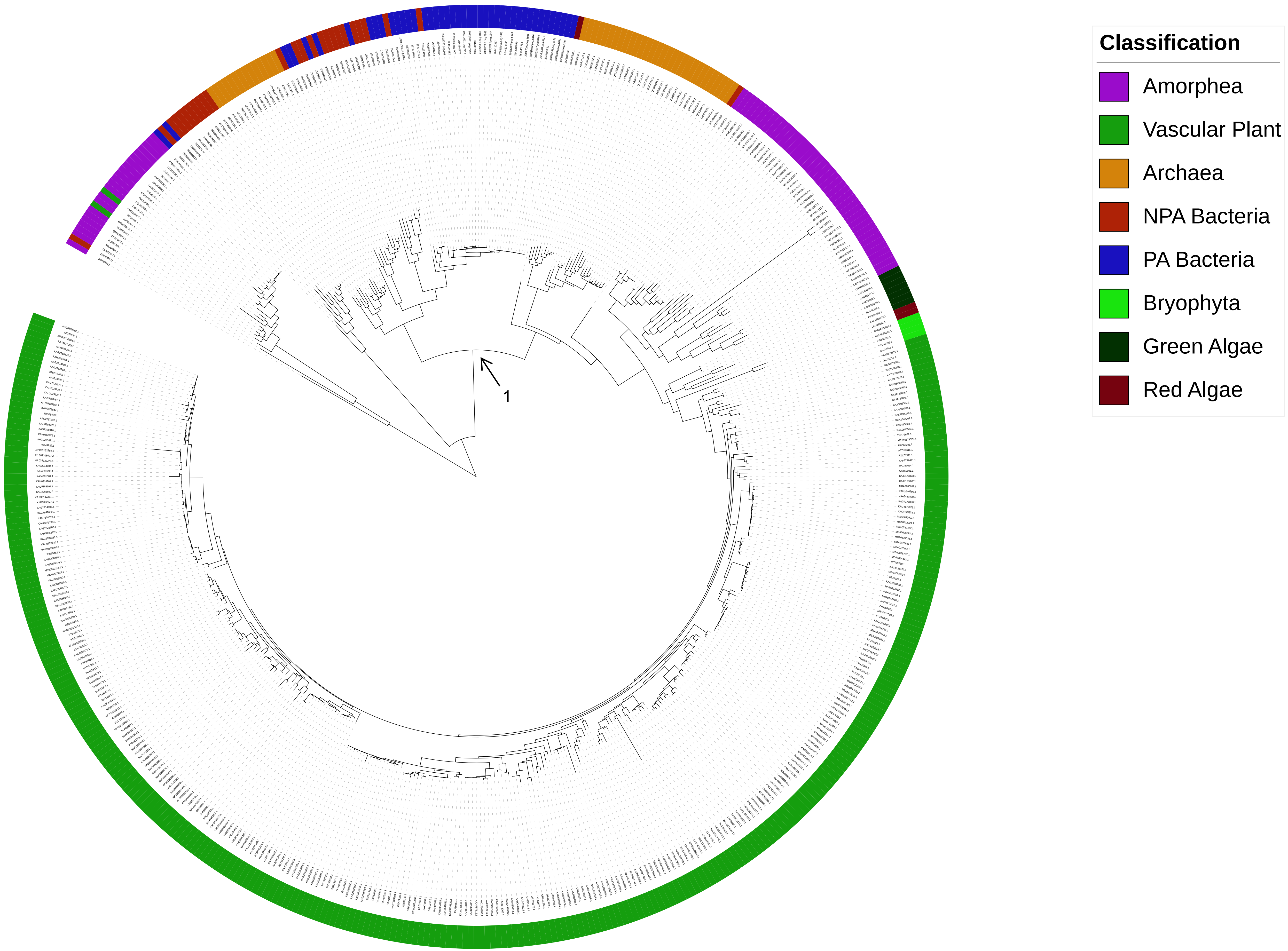

Supplemental Figure 11. Example of HGT from eukaryotes to bacteria. A phylogenetic tree that presents homologs of the SBP1 (AT4G14030) gene. The ultrafas bootstrap value of the clade that is shared by eukaryotes and bacteria is 1 - marked with an arrow

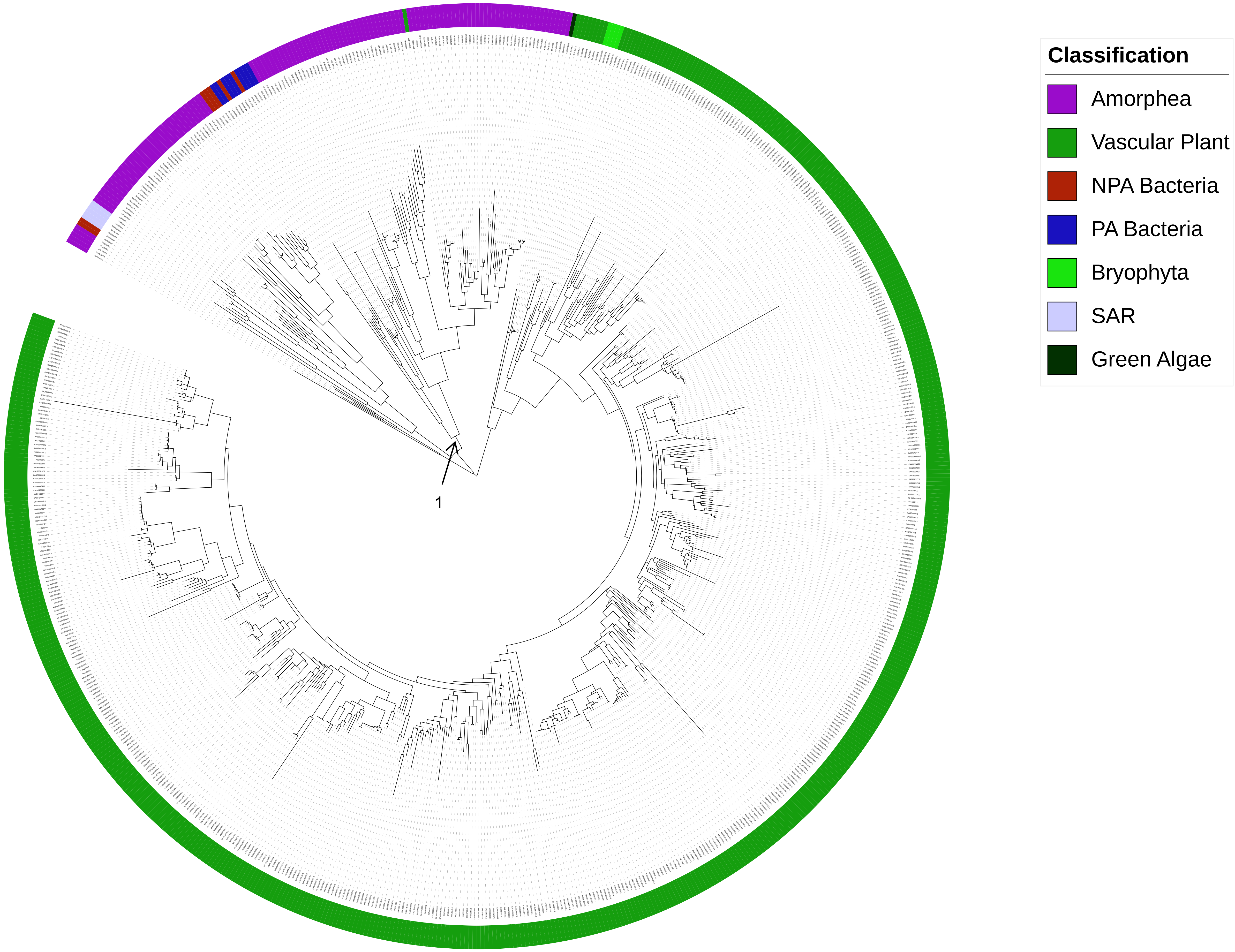

Supplemental Figure 12. Example of HGT from eukaryotes to bacteria. A phylogenetic tree that presents homologs of the MIOX4 (AT4G26260) gene. The ultrafas bootstrap value of the clade that is shared by eukaryotes and bacteria is 1 - marked with an arrow

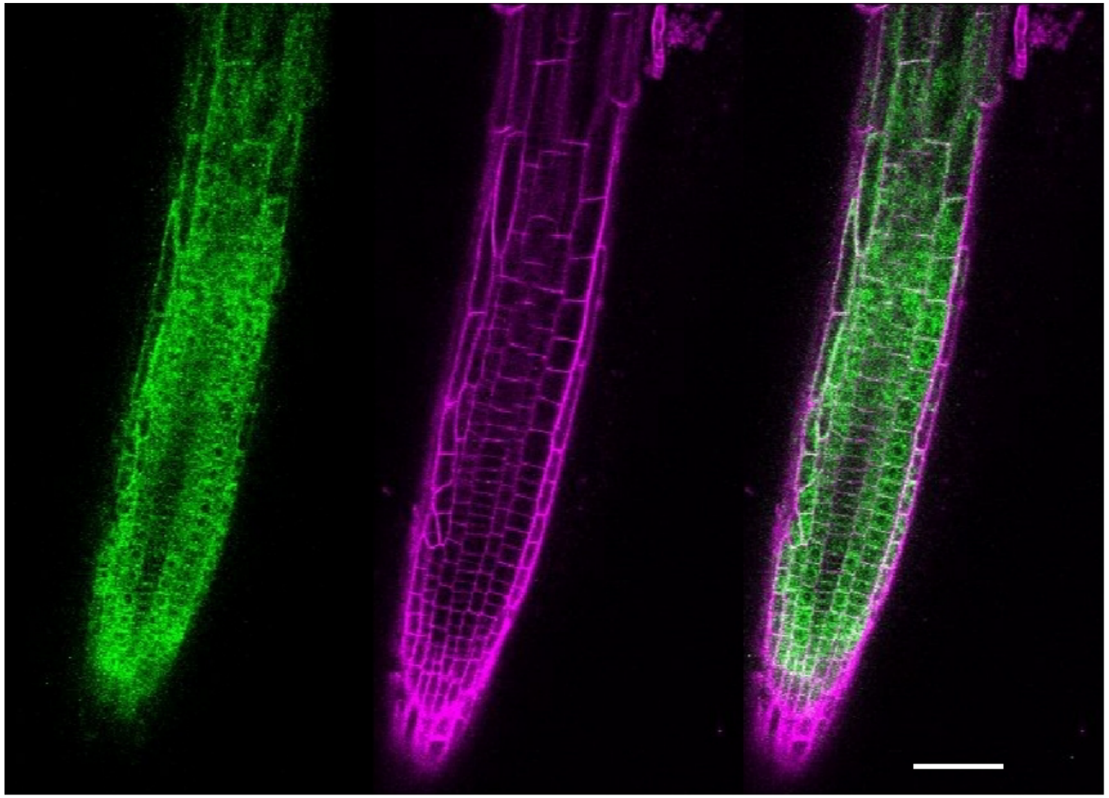

p35S:lfDET2-NG

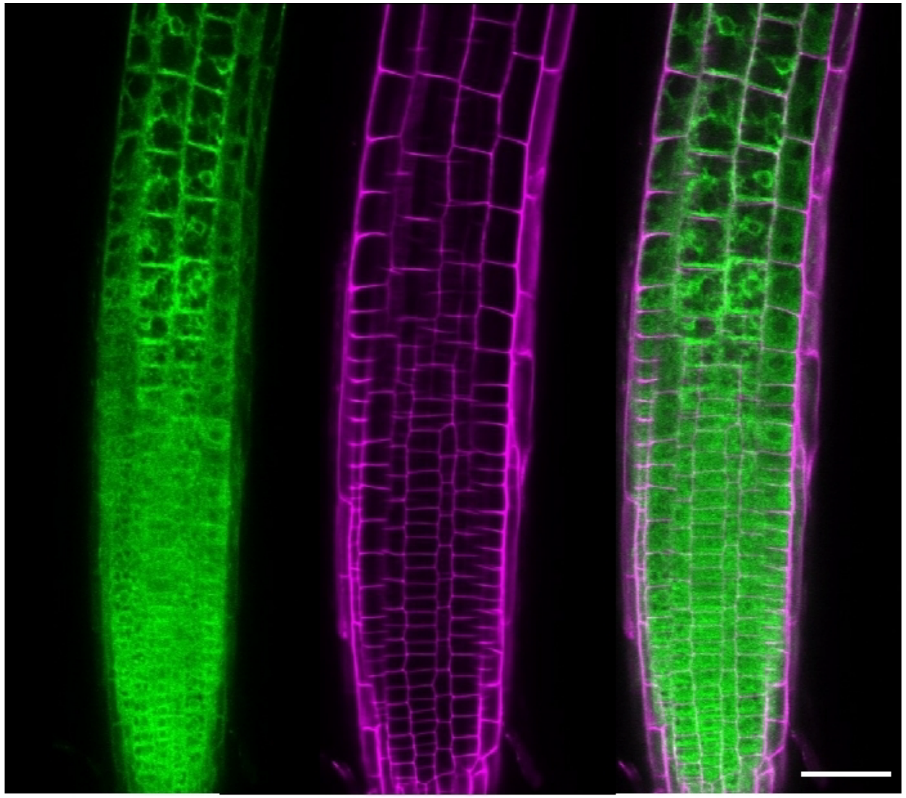

pDET2:atDET2-NG

Supplemental Figure 13. Confocal images of a roots expressing lfDET2-NG in the det2 background (left) and atDET2-NG in WT background (right). NG is shown in green and propidium iodide (PI) that marks cell borders in magenta
